# Building Research Capacity for Impact in Applied Health Services Research Partnerships Comment on "Experience of Health Leadership in Partnering With University-Based Researchers in Canada – A Call to "Re-imagine" Research"

**DOI:** 10.15171/ijhpm.2020.11

**Published:** 2020-02-02

**Authors:** Jo Cooke

**Affiliations:** ^1^NIHR Collaboration and Leadership in Applied Health Research and Care for Yorkshire and Humber (CLAHRC YH), Sheffield, UK.; ^2^Sheffield Teaching Hospitals NHS Foundation Trust, Sheffield, UK.

**Keywords:** Research Capacity Building, Co-production, Research Impact, Research Networks, Research Collaborations, UK

## Abstract

Bowen and colleagues ask us to re-imagine how to conduct research in academic-practice partnerships, and to develop capacity in the applied research *and* health workforce to do this. This commentary reinforces their messages, and describes a framework of research capacity development for impact (RCDi) which emphasizes active and continuous experiential learning within research partnerships. The RCDi framework includes the need to focus on multiple levels in the collaboration architecture, and describes principles of working that aims to increase impact on services, and learning opportunities for all partners.

The paper by Bowen and colleagues^1^ highlights the importance of developing strong collaborative partnerships between health service researchers and the end users of the research to ensure impact. It calls us to ‘re-imagine’ the types of methodologies adopted, and to blur boundaries between knowledge production and quality improvement. Importantly, it identifies a need to develop capacity in the applied research *and* health workforce to do this. This commentary reinforces their messages and extends them by describing an approach where research capacity can be developed in both academia and practice. It is based on our experience of academic leadership in a funded research collaboration in the United Kingdom based in an National Health Service (NHS) Trust. We suggest that research capacity development (RCD) should be embedded in such ‘on-going’ research collaborations, and should be characterised by ‘learning by doing’ activity, and RCD interventions at multiple levels of the collaboration, and across sectors shaped by principles of RCD for impact (RCDi).

This commentary is based on learning we have developed in over a decade of undertaking RCDi in a complex research partnership called a Collaboration and Leadership in Applied Health Research and Care (CLAHRC). Our CLAHRC included 14 NHS Trusts, three health charities, six universities and four Local Government Authorities, and served a population of 5 million. This CLAHRC was one of 13 funded in England by the National Institute for Health Research to accomplish three objectives: to develop a programme of relevant applied research; to support research implementation and knowledge mobilization, and develop capacity within people and organizations to do both^2^ in order to address the research-practice gap.

True to the experimental nature within the CLAHRC community,^3^ our RCDi approach was developed iteratively by using the approach described here to guide policy, practice, RCD interventions and research project decisions in real time, supported by internal evaluations, and research evidence. The starting point was an evidence-based framework,^4^ which was adapted over time. The resulting RCDi framework is represented in Figure. It aims to produce skills in people, and inform processes in organizations and wider research health systems to plan, develop, and execute impactful research. The RCDi framework incorporates mode 2 research (where knowledge is produced where it is to be used), promotes creative partnerships, and aims to trigger mechanisms that support RCD based on a realist review.^5^ Capacity building is seen not an end in itself, but a means to an end: that is it is designed to develop and produce research that is used and impactful.

**Figure:**
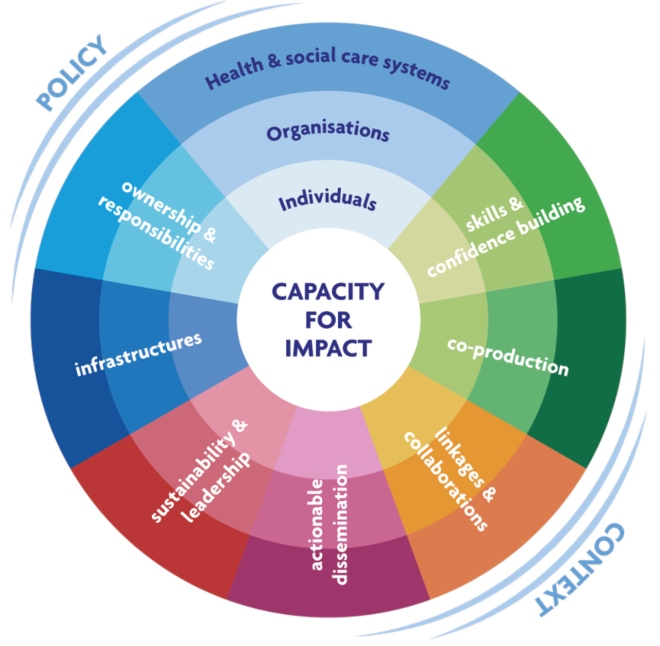
A Framework for RCDi. Abbreviation: RCDi, research capacity development for impact.

It has two features: structural levels, and cross cutting principles or ‘ways of working’ in the context of health and research policy.

## The Structural Levels

The structural levels encompass the complex architecture of on-going research partnerships such as CLAHRCs. They include individuals, organizations (both academic and care providers) and systems through which these partnerships operate.^6,7^

Systematic reviews recognise that RCD interventions should operate at multiple levels^5,8^ and be planned concurrently at different levels to be effective.^6^ So, for example, interventions aimed at individual RCD such as internships, fellowships and secondments, should be enabled by organizational and cross organizational processes that encompass protected research time, budget planning, and resource management and exchange. It also includes career pathways that cross-organizational boundaries that are enabled by human resource systems, mentorship and coaching. A lack of institutional support is often a barrier to RCD for individuals^9^ and adopting a multilevel approach to planning is essential to overcome such barriers.

Cross organization and systems levels RCDi planning includes ‘learning by doing’ within research collaboration itself. This experiential learning enables adaptation and change across sectors and within systems.^7^ In this way the collaboration itself acts as an RCD intervention, in that it provides learning for all participants as the research programme is undertaken. Activities that enable this include learning to set priorities and negotiate win-win solutions where all partners feel that they have benefitted from decisions made, promoting trust and reciprocity across organizations and sectors.^2^

## The Principles

The principles of the framework represent guidance that should shape and inform RCDi plans and RCD interventions at each level, so they are ‘hard-wired’ or integral to the partnership process. The CLAHRC provided learning opportunities within the research and knowledge mobilisation projects for all partners, as well as developing placements for academics from diverse academic disciplines (for example health economics and design) to work into NHS organizations. RCD interventions for NHS colleagues included secondments, fellowships and research internships. An example of how such opportunities were shaped by the RCDi framework is given in Box 1 on a research internship scheme for individuals, and in a community of practice (CoP) in Box 2 at an organizational level.

**Box 1. Pre-masters Research Internship**

These focussed on providing ‘learning by doing’ opportunities for NMAHPs. Practitioners were funded to have protected time away from clinical duties to work on a project with CLAHRC academics. The internship course offered workshops around the principles in the framework, and interns were asked to develop learning diaries of how their experience helped to extend their knowledge-based on these principles. For example: new skills developed; the networking opportunities it provided; experiences or observations around coproduction, and use of actionable outputs. Mindful of structural levels in the RCDi framework, the internship work plan and application was ‘signed off’ by their clinical manager, and we conducted workshops with managers and interns to support protected time arrangements, career planning, and how newly acquired skills/knowledge could be useful for practice, thus helping to support influence on service and workforce planning.

Abbreviations: NMAHPs, Nurses, Midwives and Allied Health Professionals; CLAHRC, Collaboration and Leadership in Applied Health Research and Care; RCDi, research capacity development for impact.

**Box 2. The ACORN Community of Practice**

The ACORN CoP aims to mobilise and develop knowledge around RCD within service provider organizations. At the time of print ACORN comprises of 12 NHS organizations and one Local Authority. Members include R&D managers and Clinical Leads in NMAHPs. Each organization undertakes a reflection of their research strategy using a self-assess tool that was developed from the original RCD Framework and adapted by them. These findings are shared in the group to support forward planning.

The ACORN group meets three times a year, and undertakes a programme of work decided by them. The group shares policy documents such as R&D strategies, job descriptions, training guides etc. Members also undertake workshops, and knowledge exchange events together, for example, about how to develop and assess the impact of academic-practice posts, the development of clinical research careers for NMAHPs, and developing and expanding networks into academia. The group also delivers service development projects. One example of this being the VICTOR project which aims to describe the impact of undertaking research in service provider organizations (see http://clahrc-yh.nihr.ac.uk/victorimpact).

Abbreviations: ACORN, Addressing Capacity in Organizations to do Research Network; CoP, community of practice; NMAHPs, Nurses, Midwives and Allied Health Professionals; RCD, research capacity development; NHS, National Health Service; R&D, Research and development; VICTOR, Visible ImpaCT Of Research.

### Skills and Confidence Building

The RCDi skills should encompass a range of evaluation methodologies and tradition research skills,^9^ but importantly, they should include boundary spanning skills and relationship brokering experience.^10^ This includes developing skills and confidence in working across different organizational, professional and academic discipline boundaries, and between different sectors. It encompasses developing an understanding of how partner organizations function, the language they use, and their values and approaches that are important to them. Such skills have been highlighted by Bowen et al^1^ as important, and call for us to re-imagine how to do this. Rather than through formal training of researchers suggested by them, we have found this is best achieved through experiential learning for both researchers and practitioners/policy-makers in research projects, in placement/secondment opportunities, and CoP, supported by mentorship and ‘leading by example.’ CoPs in the CLAHRC have included a clinical focus, for example in developing and then using a patient reported outcome measure (PROM) assessing the quality of life for people using mental health services. This measure blends knowledge from different stakeholders and is now an actionable tool being used in a range of organizations. Its continued use is supported by a CoP which includes members of the research team and service providers and policy-makers from diverse sectors. Not only does this CoP help explore how to use this measure in different contexts and client groups, it shares and develops this knowledge together, building confidence in participants and networking skills. It has also developed further research questions, for example, extending the work into learning disabilities, and with different cultural groups. Thus skills and knowledge were developed in the research team and services, as they worked together to produce more research and expand the use of the actionable tool in practice.

Structure and governance systems such as collaborative agreements and memoranda of understanding can formalize this iterative learning context.

### Co-production

This principle embraces the ‘mode 2’ knowledge production, where knowledge is created in the context of its application through collaboration, leading to action and impact.^11^ It also enables the blurring of boundaries between applied research and knowledge mobilization called for by Bowen et al. Our CLAHRC defined coproduction as ‘activity that engages the right people (service users, practitioners, NHS and care managers, and academics from a range of disciplines) to make decisions and support the conduct of projects and activities on issues that are important and matter to them.’^2^ We have used Delphi and other consensus techniques as well as workshops discussions to set research priorities^2^ to do this. We have found that co-creative practices, using design approaches can be helpful for power sharing.^12^ One research theme, for example, developed a series of ‘Getting Research into Practice’(GRiP) projects to work with clinical teams and services users on projects using co-creative design methods with them. Clinical teams applied to the CLAHRC to undertake a GRiP project on a topic chosen by them. Diverse projects such as increasing access, and use of hepatitis C clinics; supporting adults with learning disabilities to keep warm in their own homes; and helping patients undergoing chemotherapy detect symptoms of neutropenic sepsis were delivered. A case study book of these projects, the methods adopted and outputs produced can be found online (see http://clahrc-yh.nihr.ac.uk/our-themes/translating-knowledge-into-action/2-casebook). We also provide training to support meaningful public and patient involvement for academics, practitioners and the public.

### Actionable Dissemination

Graham et al^13^ emphasise that it is an ethical duty to publish research capable of addressing the second translational gap. This RCDi framework suggests this can be achieved through ‘actionable dissemination’ often in the form of ‘actionable tools’ which are products informed by research study findings intended to change the way of thinking, promote decision-making or instigate action.^14^ They are shaped by research knowledge, use appropriate communication for the target audience, and include a prompt for action. Tracking the use of actionable tools has provided examples of immediate impact in our CLAHRC, providing visible examples of meaningful engagement. For example: the mental health PROM described earlier; a frailty measure in primary care; and a new born screening tool to identify rare diseases. These are all being used locally and nationally. Many of our impacts are described in an impact document (see https://drive.google.com/file/d/1q_qBRvuWm0E9Ri_eV0YXjC8FHQdsIIfq/view).

One actionable tool called VICTOR (the Visible ImpaCT Of Research), was developed with the ACORN (Addressing Capacity in Organizations to do Research Network) group and helps uncover and describe the impact of conducting research in an NHS organization (https://www.e-repository.clahrc-yh.nihr.ac.uk/visible-impact-of-research/).

This tool provides context specific stories that organizations can use in their annual reports, in newsletters, in external quality inspections and in feed back to the CLAHRC.

### Infrastructure

Long-term funded collaborative partnerships provide resources and architectures to support RCDi. Allocating and releasing resources is an important mechanism to enable RCD.^4,8^ This can be made tangible through allocation of research grant budgets to fund activities and time of both academics and policy-makers/practitioners. It can also be made possible through ‘matched funding’ arrangements where all types of collaborating organizations offer resource such as time, facilities or fee waivers into the collaboration free of charge to the research partnership. The relatively long-term funding period of the CLAHRC (5 years for each term) has provided an on-going academic and practice infrastructure^15^ and opportunities for iterative learning embedded in special interest groups, steering and advisory groups^2^ and CoPs.

### Linkages and Collaborations

Long-term research partnership can provide opportunities for synergy between stakeholder groups, which is an important mechanism for RCD.^5^ Diverse disciplines exercising epistemological tolerance can stimulate more benefit than the sum of individual parts. Such linkages can also support knowledge mobilisation and coproduction if conducted in a meaningful manner.^7^

CLAHRCs have demonstrated an increase in social networks, partnerships and ‘relational capability’ enabled by leadership,^15^ and our experience is that these networks support successful funding applications.

### Sustainability and Leadership

Leadership is important for the cohesion and sustainability of complex research partnerships, and this was recognised as across a number of CLAHRC evaluations.^16,17^ Adopting a leadership style that encompasses flexibility, being willing to listen, and experiment to overcome challenges is important in all partnership organizations. This supports RCDi as it encourages a context that enables learning across organizational and disciplinary boundaries. Supportive and visionary leaders can introduce long-term plans for sustainability, and plan workforce development including how to maximise the use of newly acquired research skills in the research collaboration and partner organizations. Importantly, such leaders are role models for more junior members of staff which stimulates capacity.^5^

### Ownership and Responsibilities

Signalling importance of undertaking research, and making research ‘core business’ is a mechanism that promotes RCD, particularly in service-provider organizations that have high clinical demands on time. A realist review^5^ recognised the symbolic role of some RCD activities, for example protected time for conducting research in healthcare organizations. Not only does this have an instrumental role in freeing clinical staff and managers from other duties, but also demonstrates that research is important and a priority for the organization. Similarly time spent on relationship brokering by academic partners reinforces the importance of this activity by universities. In this way matched funding provided by partners embodies contribution and commitment. It legitimates time spent on research partnership work, and acts as a mechanism to ensure research aligns to partners’ organizational objectives. The on-going nature of the partnership can also mean that continued dialogue can ensure that stories of impact are shared in both directions. So for example, the PROM CoP described earlier helped services share with each other how they were using the tool, and provided fruitful collaboration for further research projects for the research team. The VICTOR tool developed by ACORN provides examples of how projects influenced organizational change, and uncovers impacts that are often unseen or a by-product^19^ of the research. The CLAHRC also produced outputs to share across the partners, in the form of newsletters, brochures, workshops, twitter feeds and films that can be used with a diverse range of stakeholders to ensure stories of impact can be shared.

## Conclusion

This paper describes a RCDi framework developed in a long-term research-practice partnership that responds to challenges identified by Bowen and colleagues. Rather than developing educational programmes for researchers traditionally based in universities, mutual joint learning within across both sectors can be achieved by embedding experiential learning within an on-going long-term partnership under visionary and brave leadership.^2^ The move from passive formal education towards active, continuous learning through participation has been supported by others.^18^ This RCDi framework can help guide such partnerships in how to achieve this across structural levels, and through principles of working that are incorporated in RCD interventions and the programme itself.

Important messages for research funders include commissioning research capacity as an inherent part of funding calls for research collaborations, include flexibility to deliver programmes in this manner, funding to develop RCD interventions at different levels, and include funded time to build trusting relationship that support ‘learning by doing’ across sectors.^10^

## Acknowledgements

The research programme was funded by the NIHR CLAHRC Yorkshire and Humber, Sheffield, UK (http://clahrc-yh.nihr.ac.uk/). The views expressed are those of the author, and not necessarily those of the NHS, the NIHR or the Department of Health.

## Ethical issues

Not applicable.

## Competing interests

Author declares that she has no competing interests.

## Author’s contribution

JC is the single author of the paper.
